# Co-parasitism by chigger mites (Trombidiformes: Leeuwenhoekiidae) and soft ticks (Ixodida: Argasidae) in Brazilian anurans, with the description of a new *Hannemania* species

**DOI:** 10.1007/s11230-026-10292-w

**Published:** 2026-07-27

**Authors:** Isabella Pereira Pesenato, Ricardo Bassini-Silva, Pedro Paulo Goulart Taucci, Nadya Carolina Pupin, Murillo Castelo Branco Monho, Giovanna Rafaeli Rezende, Maria Eduarda Barbosa de Souza-Silva, Sebastian Muñoz-Leal, Marcelo B. Labruna, Darci Moraes Barros-Battesti, Fernando de Castro Jacinavicius

**Affiliations:** 1 Departamento de Biologia Animal, Instituto de Biologia, Universidade Estadual de Campinas (UNICAMP), Campinas, SP Brazil; 2 Laboratório de Coleções Zoológicas, Instituto Butantan, São Paulo, SP Brazil; 3 Laboratório de Biologia Evolutiva e Sistemática de Tetrápodes Atuais, Faculdade de Ciências, Universidade Estadual Paulista (UNESP), Bauru, São Paulo Brasil; 4 Centro de Pesquisa em Biodiversidade e Mudanças do Clima (CBioClima), Instituto de Biociências, Universidade Estadual Paulista (UNESP), Rio Claro, São Paulo Brasil; 5 Departamento de Biodiversidade e Centro de Aquicultura (CAUNESP), Instituto de Biociências, Universidade Estadual Paulista (UNESP), Rio Claro, São Paulo Brasil; 6 Departamento de Ciencia Animal, Universidad de Concepción, Chillan, Ñuble Chile; 7 Centro de Biotecnología, Universidad de Concepción, Concepción, Chile; 8 Departamento de Medicina Veterinária Preventiva e Saúde Animal, Faculdade de Medicina Veterinária e Zootecnia da, Universidade de São Paulo (FMVZ-USP), São Paulo, SP Brazil; 9 Departamento de Patologia, Faculdade de Ciências Agrárias e Veterinárias (UNESP), Reprodução e Saúde Única, Jaboticabal, SP Brazil

## Abstract

*Bokermannohyla* frogs comprise species distributed across different Brazilian biomes, several of which are currently listed as endangered species. These anurans host a diversity of parasites that may negatively affect their survival. Several tick species of the family Argasidae and chigger mites of the family Leeuwenhoekiidae exhibit host specificity toward this group. Here, we report a case of co-parasitism by argasid ticks and chigger mites in this frog genus collected in the Bahia State, Brazil, and describe a new species of *Hannemania, H. diamantinensis* **sp. nov.** Anurans were captured in Chapada Diamantina National Park, and ectoparasites were collected and identified using morphological methods. Ticks were identified as *Ornithodoros saraivai*, while chigger mites correspond to a new species. This represents the first record of *O. saraivai* in Bahia State, and the description of a new *Hannemania* species increases the number of Brazilian known species to eight.

## Introduction

The chigger mite family Leeuwenhoekiidae Womersley, 1944, is composed of five genera in the Brazilian territory with 12 valid species (Jacinavicius et al., 2018; 2021a, 2021b; Bassini-Silva et al., 2021; 2022; Pereira-Quaresma et al., 2024; Felix-Nascimento et al., 2025). The mites belonging to this family are ectoparasites of vertebrates in their larval stage. During the parasitism, these arthropods may cause a primary reaction in the hosts, known as nodular trombiculiasis (Pesenato et al., 2024). In Brazil, the species *Apolonia tigipioensis* Torres and Braga, 1938 was described causing this alteration in birds (Ornelas-Almeida et al., 1938; Bassini-Silva et al., 2025) and humans (Carneiro, 1949) and the genus *Hannemania* Oudemans, 1911, causing trombiculiasis in amphibians (Hatano et al., 2007; Bassini-Silva et al., 2021).

On the other hand, the family Argasidae Murray, 1877 is represented by four genera in Brazil, with the genus *Ornithodoros* Koch, 1844 comprising the largest species diversity among all (Dantas-Torres et al., 2019). The species from this genus are ectoparasites in all life stages known to parasitize amphibians (Barros-Battesti et al., 2015), reptiles (Venzal et al., 2013; 2015), bats (Muñoz-Leal et al., 2018) and humans (Dantas-Torres et al., 2022; Nogueira et al., 2022).

The genus *Bokermannohyla* Faivovich, Haddad, Garcia, Frost, Campbell, and Wheeler, 2005 is represented by 30 endemic Brazilian species, occurring in the Atlantic Forest, Caatinga and Cerrado biomes (Frost, 2026). In this group, several species have declining populations ranging from least concern to critically endangered (IUCN, 2025). Faivovich et al. (2009) reported the presence of trombiculid mites during the description of *Bokermannohyla juiju* Faivovich et al., 2009, and to date, no additional records of parasitism have been documented for this genus.

In the present study, we report a case of co-parasitism involving these two distinct families of ectoparasites in two Brazilian treefrog species from the genus *Bokermannohyla*, being one of them endangered (IUCN, 2025), along with the description of a new *Hannemania* species.

## Materials and methods

**Fig. 1:**
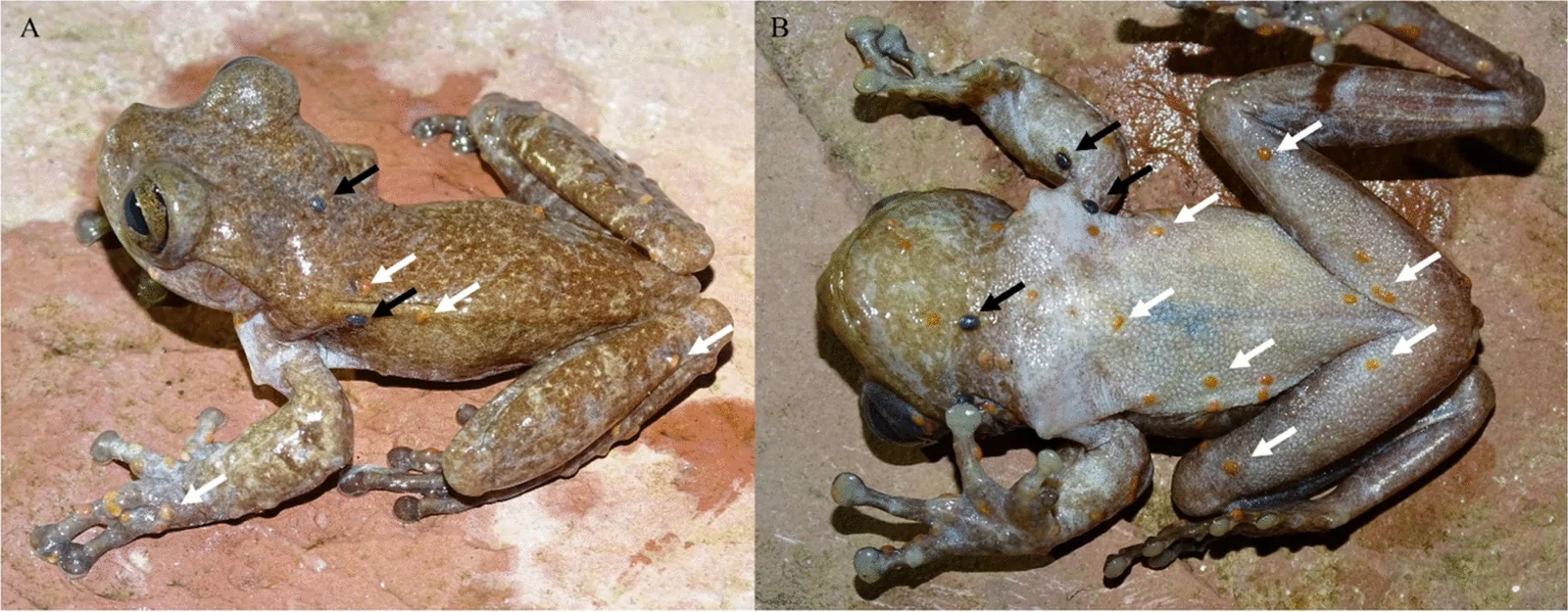
**A** Dorsal view of the co-infestation by *Hannemania diamantinensis* **sp. nov.** and *Ornithodoros saraivai* on a specimen of *Bokermannohyla juiju*; **B** ventral view of the same individual. Black setae point to the soft ticks and white setae point to the chigger specimens

Four soft ticks and seven chigger mites were collected from one frog of the species *B. juiju* (CFBH 45219; 2 larvae of soft ticks and 5 larvae of chigger mites) and one of the species *Bokermannohyla oxente* Lugli & Haddad, 2006 (CFBH 45220; 2 larvae of soft ticks and 2 larvae of chigger mites) at the Rio Ribeirão do Meio at the trail for the Sossego Waterfall, Parque Nacional da Chapada Diamantina, Lençóis municipality, Bahia state, Brazil (12° 35′ 36″ S, 41° 24′ 42″ W). The specimens were deposited in the Amphibian collection Célio Fernando Baptista Haddad (CFBH) at the Instituto de Biociências of the Universidade Estadual Paulista (UNESP), Campus Rio Claro, Brazil. The collection of the specimens was conducted as described in Taucce et al. (2015). The mites were found throughout the hosts’ bodies, whereas the ticks were found mainly on the back and legs (Figure 1A, B).

After the examination of the amphibians, the samples were collected and stored in absolute ethanol and sent to the Acarological Collection at the Instituto de Biologia of the Universidade Estadual de Campinas (UNICAMP). Part of the samples was diaphanized in lactic acid and slide-mounted in Hoyer’s medium, following the protocol described by Krantz & Walter (2009) and part of this material was prepared for Scanning Electron Microscopy (SEM), using the Digital Scanning Microscope FEI, Quanta 250, at the Laboratório de Biologia Estrutural e Funcional, Instituto Butantan, São Paulo, Brazil.

The ticks were identified by following the key provided by Dantas-Torres et al. (2019) and original species description by Muñoz-Leal et al. (2017). The chigger specimens were identified at the genus level using the Neotropical and Nearctic keys published by Brennan & Goff (1977) and subsequently identified to the species level by comparing them to the original descriptions and redescriptions. We used the nomenclature proposed by Grandjean (1939) with adaptations by Kethley (1990) for the dorsal opisthosomal setae and setae on the prodorsal sclerite and Grandjean (1935, 1947) for the specialized setae on the legs and palps. Also, additional nomenclatures were based on the glossary proposed by Goff et al. (1982).

Morphological characters and the measurements of types were performed using a Leica Microscope DM4000B, with a camera lucida. Extended focal range images were compiled with the Leica Application Suite version 2.5.0 software. The images were prepared with Adobe Photoshop v.13.0 and Inkscape v.1.4.3.

Measurements are given in micrometers (Tables 1, 2, 3, 4). The length measurement of σ I is the average of all solenidia observed in the genu of leg I. All abbreviations and symbols used in the tables are listed below: AW = distance between the bases of *ve* setae; PW = distance between the bases of se setae; SB = distance between the trichobothridial (*si*) bases; ASB = distance from bases of trichobothria (*si*) to extreme anterior margin of prodorsal sclerite; PSB = distance from bases of trichobothria (*si*) to extreme posterior margin of prodorsal sclerite; SD = ASB + PSB; P-PL = distance from bases of se setae to extreme posterior margin of prodorsal sclerite; AP = distance between the bases of *ve* and *se*; *vi* = anteromedial seta; *ve* = external vertical setae; *se* = external scapular setae; *si* = internal scapular setae (trichobothria); *1a* = anterior sternal setae; *3a* = posterior sternal setae; DMIN = minimum length of dorsal opisthosomal setae; DMAX = maximum length of dorsal opisthosomal setae; VMIN = minimum length of ventral idiosomal setae; VMAX = maximum length of ventral idiosomal setae; Cx I – length of coxa I; Tr I – length of trochanter I; Fe I – length of femur I; Ge I – length of genu I; Ti I – length of tibia I; Ta I (L) – length of tarsus I; Ta I (W) – width of tarsus I; Cx II – length of coxa II; Tr II – length of trochanter II; Fe II – length of basifemur II; Ge II – length of genu II; Ti II – length of tibia II; Ta II (L) – length of tarsus II; Ta II (W) – width of tarsus II; Legend: Cx III – length of coxa III; Tr III – length of trochanter III; Fe III – length of basifemur III; Ge III – length of genu III; Ti III – length of tibia III; Ta III (L) – length of tarsus III; Ta III (W) – width of tarsus III; I = length of leg I (trochanter to tarsus); II = length of leg II (trochanter to tarsus); III = length of leg III (trochanter to tarsus); Legend: σ I, σ II, σ III = length of the solenidia on Ge I–III; κ I, κ’ I = length of microseta on Ge and Ti I, respectively; φ’ I, φ” I = length of the solenidia on Ti I; ω I = length of solenidion on Ta I; ε I = length of famulus on Ta I; ζ’ I = length of dorsal eupathidium on Ta I; z = length of companion seta on Ta I; ζ’’ I = length of subterminal eupathidium on Ta I; φ’ II, φ” II = length of the solenidia on Ti II; ω II = length of solenidion on Ta II; ε II = length of famulus on Ta II; ζ II = length of subterminal eupathidium on Ta I; φ III = length of the solenidia on Ti III.

**Table 1:** Standard measurements of the types of *Hannemania diamantinensis* Pesenato, Bassini-Silva, and Jacinavicius, **sp. nov.**

	AW	PW	SB	ASB	PSB	SD	P-PL	AP	*ve*	*se*	*si*	*1a*	*3a*	DMIN	DMAX	VMIN	VMAX
Holotype	135	172	77	82	69	151	97	44	117	199	203	33	27	23	33	18	21
Paratype I	133	171	76	80	68	150	95	44	116	197	200	32	26	22	31	17	20
Paratype II	134	172	76	82	69	151	96	44	117	199	201	33	27	23	32	18	21
Mean	134	171	76	81	68	150	96	44	116	198	201	32	26	22	31	17	20

**Table 2:** Standard measurements of the types of *Hannemania diamantinensis* Pesenato, Bassini-Silva, and Jacinavicius, **sp. nov.**

	Cx I	Tr I	Fe I	Ge I	Ti I	Ta I (L)	Ta I (W)	Cx II	Tr II	Fe II	Ge II	Ti II	Ta II (L)	Ta II (W)
Holotype	71	26	71	53	71	119	20	81	26	38	44	58	86	19
Paratype I	70	25	69	52	70	117	19	80	24	37	43	56	85	18
Paratype II	71	26	71	53	71	118	19	81	25	38	44	57	86	19
Mean	70	25	70	52	70	118	19	80	25	37	43	57	85	18

**Table 3:** Standard measurements of the types of *Hannemania diamantinensis* Pesenato, Bassini-Silva, and Jacinavicius, **sp. nov.**

	Cx III	Tr III	Fe III	Ge III	Ti III	Ta III (L)	Ta III (W)	I	II	III
Holotype	64	45	67	51	71	101	18	411	333	399
Paratype I	62	44	66	50	71	100	18	403	325	393
Paratype II	64	45	67	51	71	101	18	410	331	399
Mean	63	44	66	50	71	100	18	408	329	397

**Table 4:** Standard measurements of the types of *Hannemania diamantinensis* Pesenato, Bassini-Silva, and Jacinavicius, **sp. nov.**

	σ I	κ I	φ’ I	φ’’ I	κ’ I	ω I	ε I	ζ’ I	z	ζ’’ I	σ II	κ II	φ’ II	φ” II	ω II	ε II	ζ II	σ III	φ III
Holotype	14-25	3	24	22	4	28	3	22	20	8	25	4	22	22	27	3	10	36	30
Paratype I	12-22	3	23	21	4	26	3	21	19	8	24	4	20	21	26	3	8	35	30
Paratype II	13-25	3	24	22	4	28	3	22	20	8	25	4	21	21	27	3	10	36	30
Mean	18	3	23	21	4	26	3	21	19	8	24	4	20	21	26	3	9	35	30

## Results

### Morphological analysis

**Fig. 2:**
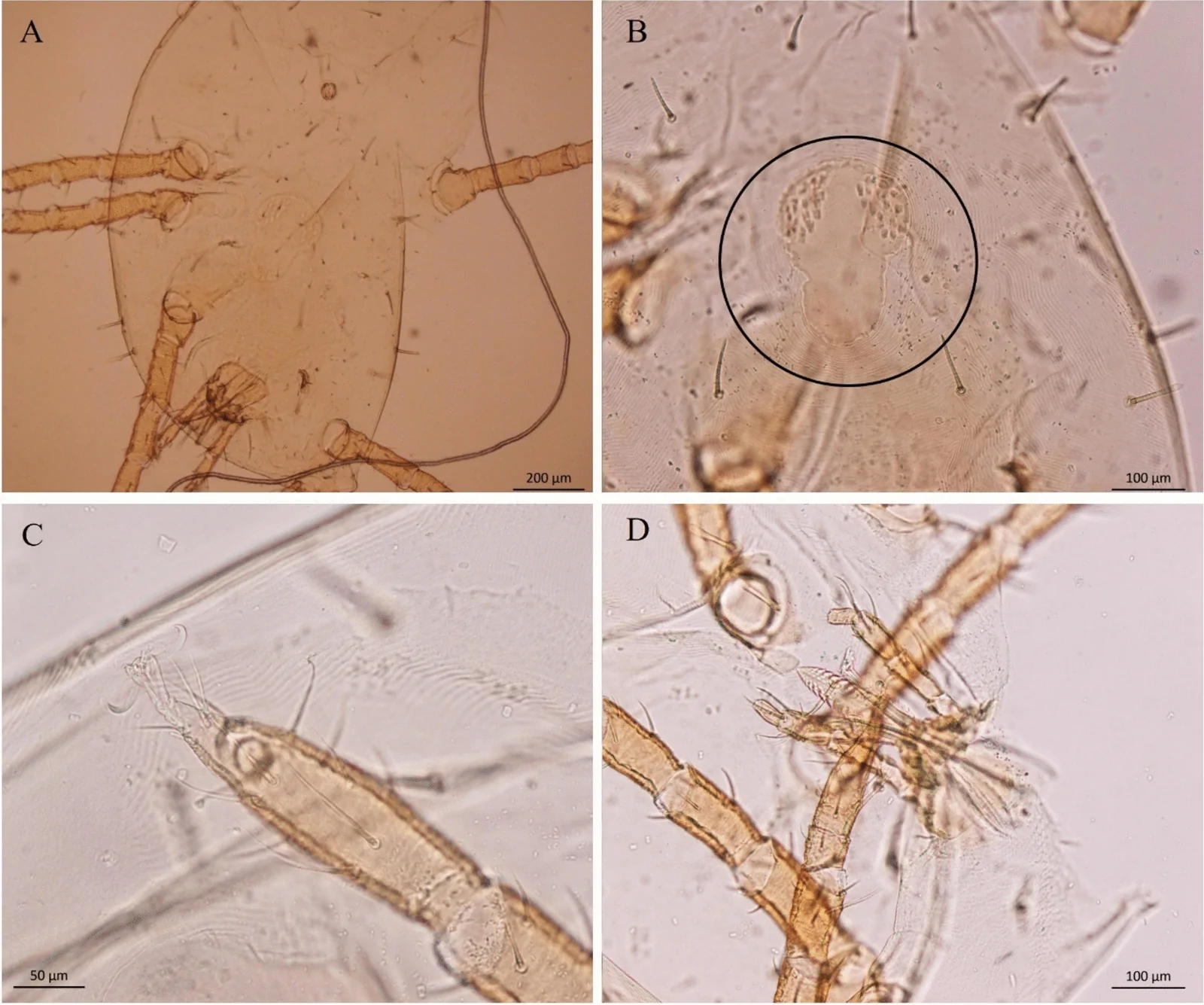
*Ornithodoros saraivai* larva: **A**. dorsal view; **B** dorsal plate (circle); **C** tarsus I; **D** gnathosome

All the tick specimens were identified as larvae of *Ornithodoros saraivai* Muñoz-Leal et al., 2017 (Figure 2A–D). The main diagnostic characters observed included a pyriform dorsal plate, 16 dorsal setae, 11 pairs of ventral setae, a pentagonal basis capituli, a dental formula of 2/2 proximally and 3/3 distally, and coxae I–III each bearing two ventral setae and one ventral spur. The specimens are deposited at the ZUEC-ACA collection under the numbers ZUEC-ACA 68A (2 larvae) and 69A (2 larvae). This is the first record of this tick species in Bahia State, and we also report two new host records: *B. juiju* and *B. oxente*.

## Taxonomy

**Family Leeuwenhoekiidae Womersley, 1944**

***Hannemania*** **Oudemans, 1911**

**Type species.** *Heterothrombidium hylodeus* Oudemans, 1910, by original designation

**Fig. 3:**
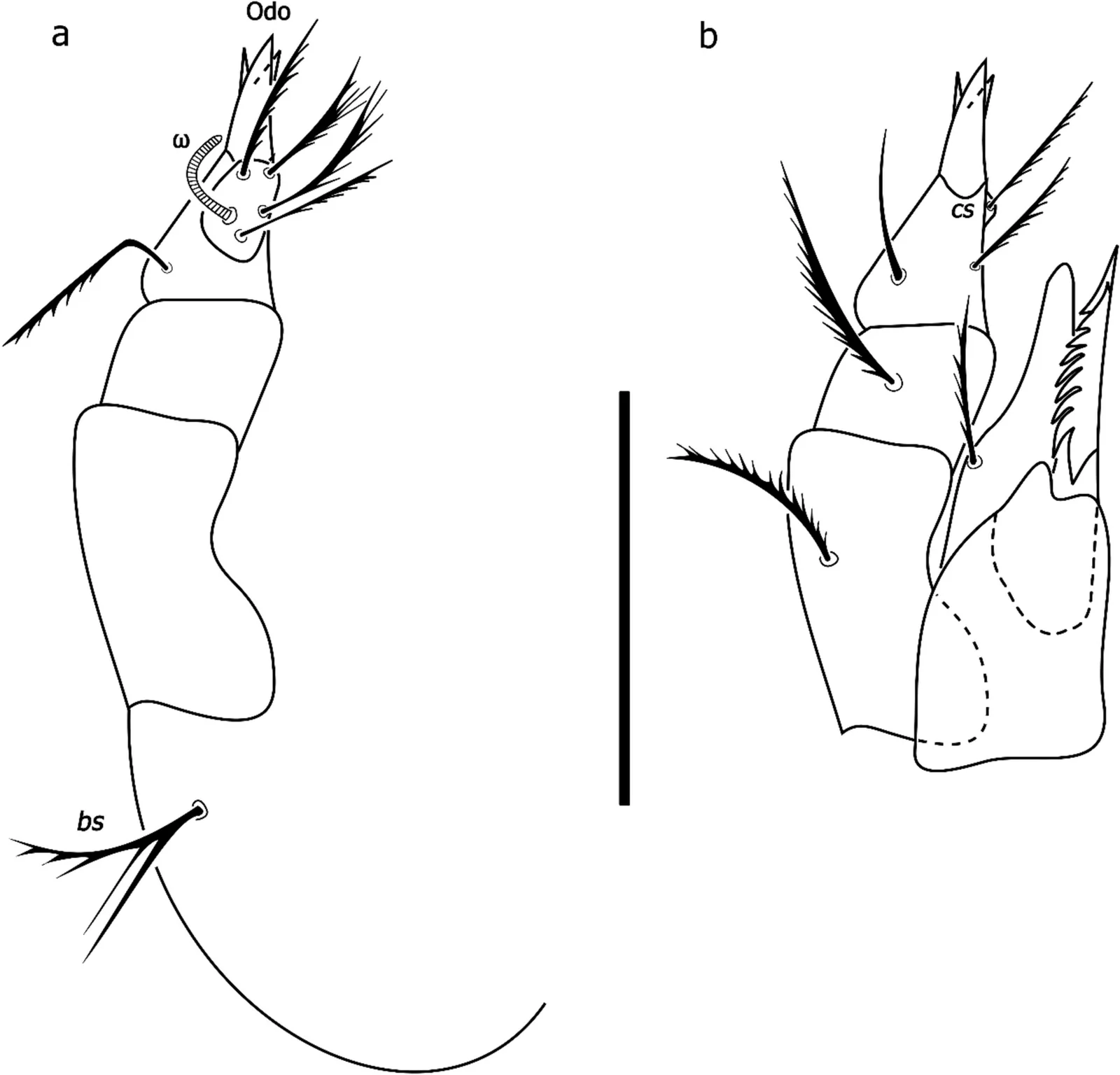
*Hannemania diamantinensis* Pesenato, Bassini-Silva, and Jacinavicius, **sp. nov.** (holotype); **a** ventral view of gnathosoma; **b** dorsal view of palp. Symbols: ω = solenidion on palptarsus; Odo = odontus; *cs* = adoral setae; *bs* = subcapitular setae. Scale bar: 50 μm

**Fig. 4:**
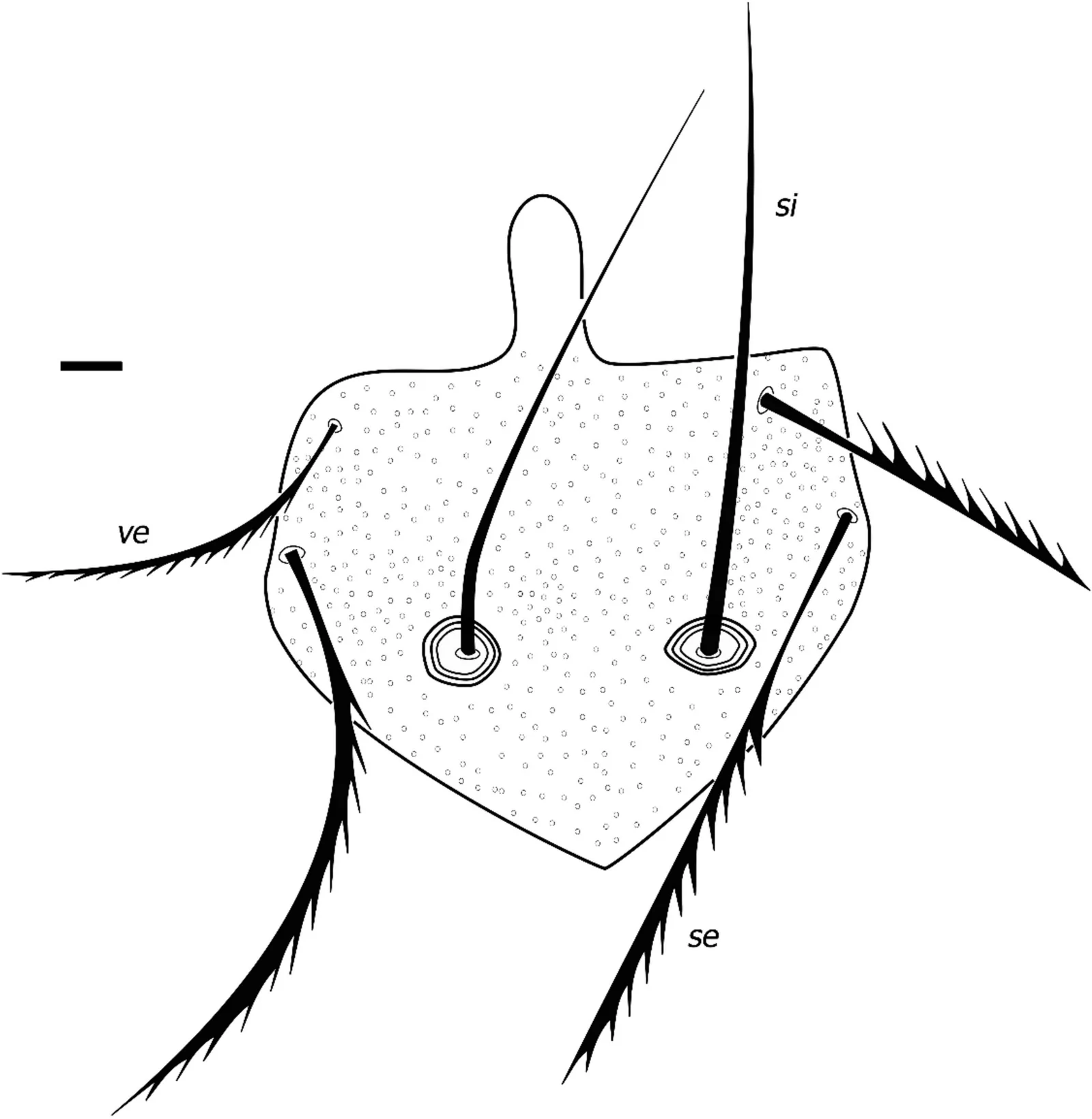
*Hannemania diamantinensis* Pesenato, Bassini-Silva, and Jacinavicius **sp. nov.** (holotype); prodorsal sclerite. Symbols: *ve* = external vertical setae; *se* = external scapular setae; *si* = internal scapular setae (trichobothria). Scale bar: 20 μm

**Fig. 5:**
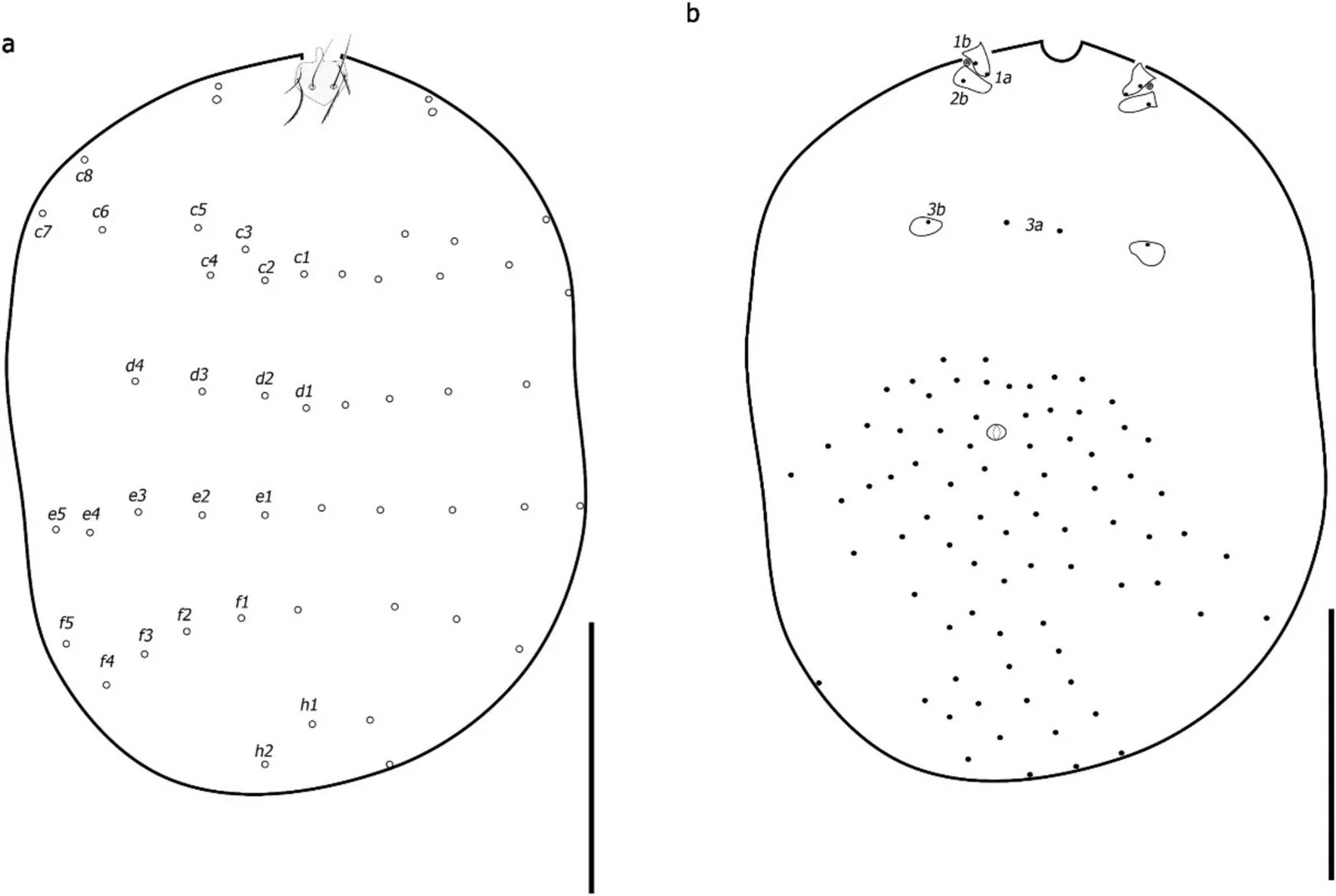
*Hannemania diamantinensis* Pesenato, Bassini-Silva, and Jacinavicius **sp. nov.** (paratype); **a** dorsal view of idiosoma; **b** ventral view of idiosoma. Solid circles = ventral setae; open circles = dorsal setae; symbols: c_1_–c_6_ = C row setae; d_1_–d_4_ = D row setae; e_1_–e_5_ = E row setae; f_1_–f_3_ = F row setae; h_1_–h_2_ = H row setae *1a* = anterior sternal setae; *3a* = posterior sternal setae; *1b* = coxal field I seta; *2b* = coxal field II seta; *3b* = coxal field III seta. Scale bars: 100 μm

**Fig. 6:**
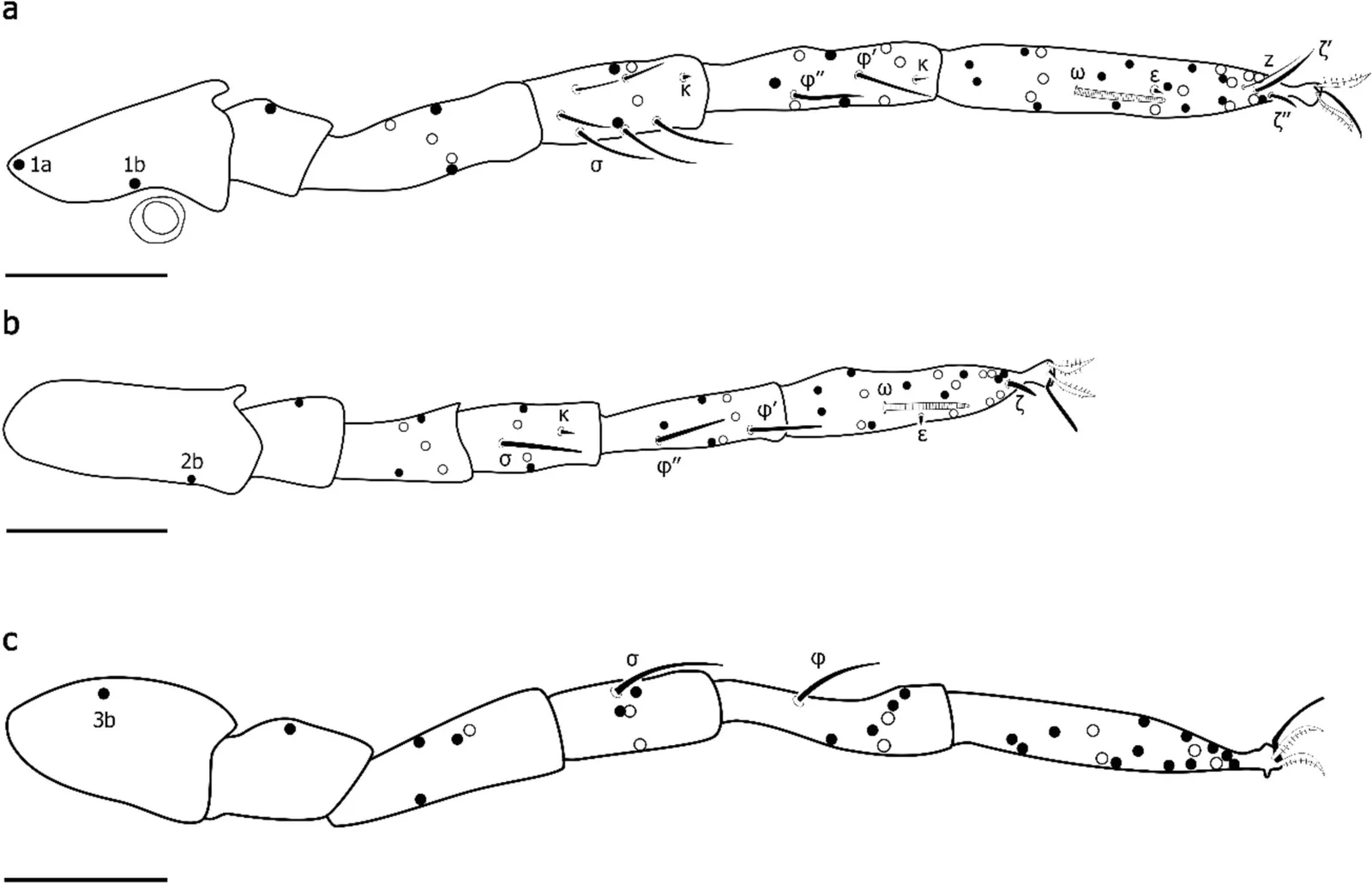
*Hannemania diamantinensis* Pesenato, Bassini-Silva, and Jacinavicius **sp. nov.** (paratype); (holotype); **a** Leg I; **b** Leg II; **c** Leg III. Solid circles = ventral leg setae; open circles = dorsal leg setae; symbols: ζ’ = dorsal eupathidium on Ta I; ζ” and ζ = subterminal eupathidium on Ta I and II; ω = solenidion on Ta I and II; σ = solenidia on Ge I–III; κ = microsetae on Ge and Ti I; φ, φ’, φ” = solenidia on Ti I–III; ε = famulus on Ta I and II; z = companion seta on Ta I; 1a = anterior sternal setae; 1b = coxal field I seta; 2b = coxal field II seta; 3b = coxal field III seta. Scale bars: a–c 50 μm

***Hannemania diamantinensis*** Pesenato, Bassini-Silva and Jacinavicius**, sp. nov.** (Figures 3, 4, 5, 6, Tables 1, 2, 3, 4)

**Diagnosis.** Palpgenu with single branched seta; dorsal and ventral palptibial setae branched; lateral palptibial setae nude; adoral (*cs*) setae branched; eyes without ocular plate; pair of nude flagelliform trichobothria (*si*); middle of posterior margin of the prodorsal sclerite with a V-shaped projection; C row with 16 setae; D row with eight setae; E row with 10 setae; F row with 10 setae; H row with four setae; 148 opisthosomal setae; 80 ventral opisthosomal setae; 6 σ on Ge I; famulus (ε) on Ta I and II each distal to solenidion (ω).

**Fig. 7:**
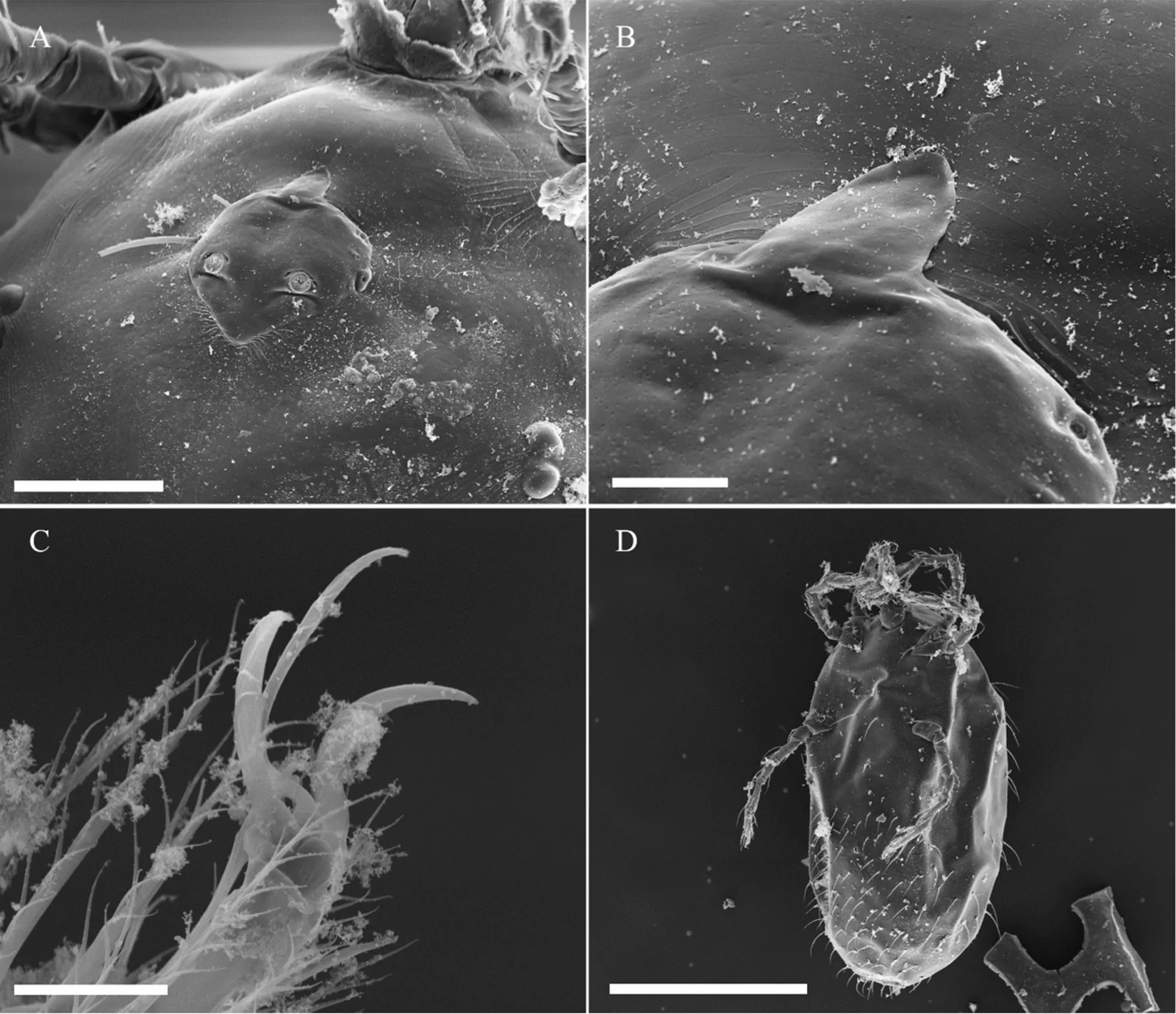
Scanning Electron Microscopy images of *Hannemania diamantinensis* Pesenato, Bassini-Silva, and Jacinavicius **sp. nov.** **A**, **B** – Prodorsal sclerite; **C** – Tarsus of Leg III; **D** – Ventral opisthosoma. *ve* external vertical setae, *se* external scapular setae, *3a* posterior sternal setae. Scales: A 50 μm, B 10 μm, C 10 μm, and D 400 μm

**Description.** Larvae (holotype and 2 paratypes). *Gnathosoma –* fPp = B/B/BNB/5B, with ω; odontus trifurcate; cheliceral blade with row of 8 outer distal teeth and a hook-shaped inner tooth; gnathobase with small punctuations, subcapitular setae (*bs*) and adoral setae (*cs*) branched (Figure 3a, b). Idiosoma – eyes 2/2, separate from prodorsal sclerite, posterior lens larger; without ocular plate, hexagonal prodorsal sclerite punctuated with nasus and lateral margins of prodorsal sclerite slightly concave, straight anterior and V-shaped projection in the middle of posterior margin (Figure 4), with pair of nude flagelliform trichobothria (*si*), 4 branched normal setae [pair of *ve* (= AL) and a pair of *se* (= PL)]; *si* > *se* > *ve* (Figure 7A, B). Opisthosoma (Figure 5a, b) with 148, dorsal opisthosoma C row with 8 pairs of irregularly placed setae, D row with 4 pairs of setae, E row with 5 pairs of setae, F row with 5 pairs of setae, H row with 2 pairs of setae, for total of 48 dorsal opisthosomal setae and 80 opisthosomal setae (19 anterior and 61 posterior to anus). One pair of sternal setae (*3a*) between coxal fields III (Figure 7D); anterior sternal setae (*1a*) located on the proximal end of coxal field I. *Legs* – femora of legs I–III entire, each leg terminates with pair of claws and claw-like empodium, with onychotriches (Figure 7C), coxal fields with few punctations; *Leg I* – two branched coxal field setae 1a and 1b (2B); trochanter 1B; femur 5B; genu 4B, 6 σ and a distal κ; tibia 8B, 2 φ and a distal κ; tarsus 24B, ω, ε, dorsal eupathidium (ζ’) with a companion seta (z) and a subterminal eupathidium (ζ”), base of famulus (ε) distal to solenidium (ω) (Figure 6a). *Leg II* – coxal field seta 2b (1B); trochanter 1B; femur 5B; genu 4B, σ and a distal κ; tibia 6B, 2 φ; tarsus 18B, ω, ε and a subterminal eupathidium (ζ), base of ε distal to ω (Figure 6b). *Leg III –* coxal field seta 3b (1B); trochanter 1B; femur 4B; genu 4B, σ; tibia 6B, φ; tarsus 16B (Figure 6c).

**Differential diagnosis.** *Hannemania diamantinensis* Pesenato, Bassini-Silva and Jacinavicius **sp. nov.** is closest to the group of *Hannemania* species that present adoral (*cs*) branched setae, odontus trifurcate, coxa III with one seta, genu II e III with one eupathidium, dorsal palptibial setae branched and lateral nude. These species include *Hannemania hylodeus* (Oudemans, 1910), *Hannemania hylae* (Ewing, 1925), *Hannemania bufonis* Loomis, 1969, *Hannemania samboni* Ewing, 1931, and *Hannemania monticola* Welbourn and Loomis, 1970. The new species can be distinguished from all of them by a combination of the following diagnostic characters: the presence of more than 100 idiosomal setae, whereas *H. hylodeus* bears only 46; a microseta on tarsus I positioned distally relative to the solenidium, in contrast to the proximal position observed in *H. hylae* and *H. bufonis*; more than three euphatidia on genu I, whereas *H. samboni* has only two; and a palptibia setal formula of BNB, differing from the BBB formula observed in *H. monticola.*

**Type material.** HOLOTYPE: larva (ZUEC-ACA 69B, IBSP 25299), Parque Nacional da Chapada Diamantina, Lençóis municipality, Bahia state, Brazil (12° 35′ 36″ S, 41° 24′ 42″ O), 19 November 2019, ex *Bokermannohyla juiju* Faivovich et al., 2009 (Anura: Hylidae) (CFBH 45219), P. Taucci and N.C. Pupin coll. PARATYPES: 4 larvae (ZUEC-ACA 69B, IBSP 25299), same data as the holotype; 2 larvae (ZUEC-ACA 68B, IBSP 25298), same locality and date as the holotype, ex *Bokermannohyla juiju* Faivovich et al., 2009 (Anura: Hylidae) (CFBH 45219) and ex *Bokermannohyla oxente* Lugli & Haddad, 2006 (Anura: Hylidae) (CFBH 45220), P. Taucci and N. C. Pupin coll.

**Type depository.** The types are deposited at the IBSP collection and ZUEC-ACA under the numbers ZUEC-ACA 68 and 69 and IBSP 25298 and 25299.

**Etymology.** The specific epithet *diamantinensis* refers to the type locality, Chapada Diamantina National Park, where the chigger was collected. The name is derived from the Portuguese word *diamantina* (“diamond”), in reference to the historical diamond mining activities in the region.

## Discussion

The occurrence of co-parasitism between different chigger species on the same host has already been reported in literature for other host groups, such as rodents (Jacinavicius et al., 2021b). In amphibians most studies have focused on endoparasites (Campião et al., 2015; Herczeg et al., 2021), lacking studies concerning their ectoparasites and their effects on their behavior and general condition. Longo et al. (2020) demonstrated a shift in the behavior of *Eleutherodactylus cooki* Thomas frogs once parasitized by ticks which affected their reproductive success and parental care. One of the individuals of *B. juiju* was heavily parasitized by both chiggers and ticks and qualitatively observed in the field to have an apparently poor body condition and reduced mobility. The observed parasitism represents a potential concern for individual health and survival, however, no causal relationship between these observations and mite parasitism can be inferred from the present study.

The species *O. saraivai* was recently described from larvae collected on the frog *Cycloramphus boraceiensis* Heyer (Cycloramphidae), and also nymphs, males and females collected in riparian habitats in an island of the state of São Paulo, southeastern Brazil (Muñoz-Leal et al., 2017). The present report expands northwards the distribution of this tick species and its host spectrum among Brazilian frogs. Another argasid species, *Ornithodoros faccinii* Barros-Battesti et al., 2015, has been associated with different frog species in southeastern Brazil and the state of Bahia (Sá-Hungaro et al., 2017). Collectively, these results point out Brazil as a unique hotspot of frog-associated argasid ticks in the world.

The description of a new *Hannemania* species raises to eight the number of species of this genus recorded in Brazil. This represents the first record of a *Hannemania* species from the state of Bahia. Previous records in Brazil include *Hannemania auiabensis* Bassini-Silva et al., 2021 from Ceará State (Bassini-Silva et al., 2021), *Hannemania hepatica* Fonseca, 1936 from São Paulo State (Fonseca, 1936), *Hannemania newsteadi* Sambon, 1928 from Mato Grosso do Sul State, *Hannemania stephensis* Sambon, 1928 from Mato Grosso State (Sambon, 1928), *Hannemania caatinguensis* Felix-Nascimento et al., 2025 from Pernambuco State (Felix-Nascimento et al., 2025), and *Hannemania achalai* Alzuet & Mauri, 1987 from Rio Grande do Sul State (Mendoza-Roldan et al., 2020). The original description of *H. hylodeus* did not include locality data.

South America is one of the world’s major hotspots of amphibian biodiversity, and Brazil is the most species-rich country globally, harboring 1,206 described anuran species distributed among 104 genera (Drummond et al., 2026; Frost, 2026). This high diversity suggests that the number of species currently recognized within the genus *Hannemania* may be underestimated, highlighting the need for more comprehensive sampling and a systematic revision of the genus.

## Data Availability

All data supporting the findings of this study are available within the paper. The slides containing the specimens identified in this article are deposited at the Acarological Collection of the Museum of Biological Diversity (MDBio), located at UNICAMP and at the Acarological Collection of the Butantan Institute and are available for examination if necessary.
